# 449. Elevated Lactate Dehydrogenase in Convalescent Severe COVID-19 Patients Reflects Delayed Clinical Recovery and Activated Th_1_ and Th_17_ Responses

**DOI:** 10.1093/ofid/ofad500.519

**Published:** 2023-11-27

**Authors:** Jinyoung Yang, Ho Cheol Jang, Min Seo Kang, Keon Young Lee, Young Ho Lee, Kyungmin Huh, Sun Young Cho, Cheol-In Kang, Doo Ryeon Chung, Kyong Ran Peck, Jae-Hoon Ko, Eui-Cheol Shin

**Affiliations:** Samsung Medical Center, Seoul, Seoul-t'ukpyolsi, Republic of Korea; Korea Advanced Institute of Science and Technology (KAIST), Daejeon, Taejon-jikhalsi, Republic of Korea; Samsung Medical Center, Seoul, Seoul-t'ukpyolsi, Republic of Korea; Samsung Medical Center, Seoul, Seoul-t'ukpyolsi, Republic of Korea; Samsung Medical Center, Seoul, Seoul-t'ukpyolsi, Republic of Korea; samsung medical center, Seoul, Seoul-t'ukpyolsi, Republic of Korea; Samsung Medical Center, Seoul, Korea, Seoul, Seoul-t'ukpyolsi, Republic of Korea; Samsung Medical Center, Seoul, Seoul-t'ukpyolsi, Republic of Korea; samsung medical center, Seoul, Seoul-t'ukpyolsi, Republic of Korea; Samsung Medical Center, Seoul, Seoul-t'ukpyolsi, Republic of Korea; Samsung Medical Center, Seoul, Seoul-t'ukpyolsi, Republic of Korea; Korea Advanced Institute of Science and Technology (KAIST), The Center for Viral Immunology, Korea Virus Research Institute, Institute for Basic Science (IBS), Daejeon, Taejon-jikhalsi, Republic of Korea

## Abstract

**Background:**

Elevated lactate dehydrogenase (LD) levels during acute phase of COVID-19 is known to reflect multiple organ injuries and clinical severity. However, clinical implication and origin of elevated LD after convalescence have not been elucidated.

**Methods:**

We prospectively enrolled non-vaccinated severe COVID-19 patients with an oxygen demand greater than FiO_2_ 0.4 and followed them up to one year after discharge. Laboratory values and modified Medical Research Council (mMRC) dyspnea scale were collected at each outpatient visit. To investigate the potential association between elevated LD levels and CD4^+^T cell activities, fluorescence-activated cell sorting (FACS) and intracellular cytokine staining (ICS) of INF-γ, TNF-α, IL-4, and IL-17A were conducted.

**Results:**

A total of 74 patients were included, of which 46 (62%) were male, the median age was 59 years (IQR 52–69), and peak FiO_2_ during hospitalization was 0.65 (IQR 0.50–0.80). At discharge, median absolute lymphocyte count (ALC) was 1.29 x10^3^/μL (IQR 0.93–1.75), median C-reactive protein (CRP) was 0.23 mg/dL (IQR 0.06–0.52), and median LD was 334 IU/L (IQR 276–450). After discharge, ALC increased (R^2^ = 0.1434, *P* < 0.001) and LD decreased (R^2^ = 0.0838, *P* < 0.001) overtime, but CRP level did not show time-dependent changes (Figure 1A-C). Increased LD (R^2^ = 0.160, *P* < 0.001) and CRP (R^2^ = 0.028, *P* = 0.024) levels were significantly associated with higher grade of mMRC scales, while ALC was not associated with mMRC scale (Fig 1D-F). To investigate the potential association between elevated LD level and CD4^+^T cell activation, serial 38 PBMC specimens from 10 patients were further investigated. CD4^+^T cells were identified using FACS gating and then stained for intracellular cytokines. The proportion of TNF-α^+^CD4^+^T cells (R^2^ = 0.547, *P* < 0.001; Fig 2A) and IL-17A^+^CD4^+^T cells (R^2^ = 0.419, *P* = 0.009; Fig 2B) after spike protein stimulation were positively correlated with LD levels, while the proportion of Treg did not.

**Figure 1.**
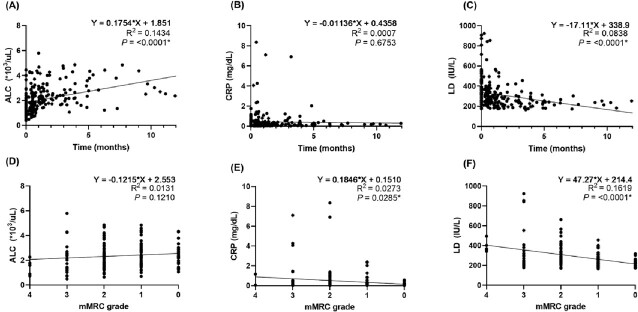
Time-dependent changes in ALC, CRP and LD levels and their association with the degree of dyspnea (mMRC scale) after discharge. (A) Linear regression with ALC and time. (B) Linear regression with CRP and time. (C) Linear regression with LD and time. (D) Linear regression with ALC and mMRC. (E) Linear regression with CRP and mMRC. (F) Linear regression with LD and mMRC. *Statistically significant. ALC, absolute lymphocyte count; CRP, C-reactive protein; LD, lactate dehydrogenase; WBC, white blood cell, mMRC, modified Medical Research Council.

**Figure 2.**
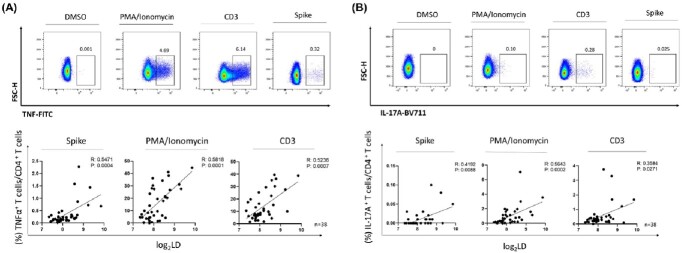
FACS gating for CD4+T cells and ICS correlation with serum LD levels after convalescence. FACS gating for CD3+CD4+T cells and ICS for TNF-α (A) and IL-17A (B) were conducted. Serum LD levels after convalescence showed significant correlations with the proportion of TNF-α+CD4+T cells (A) and that of IL-17A+CD4+T cells (B). FACS, fluorescence-activated cell sorting; ICS, intracellular cytokine staining; LD, lactate dehydrogenase.

**Conclusion:**

Elevated LD in convalescent severe COVID-19 patients reflects delayed clinical recovery and activated Th_1_ and Th_17_ responses

**Disclosures:**

**All Authors**: No reported disclosures

